# Use of high-level health facilities and catastrophic expenditure in Vietnam: can health insurance moderate this relationship?

**DOI:** 10.1186/s12913-019-4115-0

**Published:** 2019-05-21

**Authors:** Hwa-Young Lee, Juhwan Oh, Van Minh Hoang, J. Robin Moon, S. V. Subramanian

**Affiliations:** 1 JW LEE Center for Global Medicine, Seoul National University College of Medicine, 71 Ihwajang-gil, Jongno-gu, Seoul, 110-810 South Korea; 2 Department of Global Health and Population, Takemi Program in International Health, Harvard T.H. Chan School of Public Health, Boston, MA USA; 3 Center for Population Health Sciences, Hanoi University of Public Health, Hanoi, Vietnam; 4 Bronx Partners for Health Communities New York City, Bronx, NY USA; 5 Department of Social and Behavioral Sciences, Harvard T.H. Chan School of Public Health, Boston, MA USA; 6 Harvard Center for Population and Development Studies, Harvard T.H. Chan School of Public Health, Boston, MA USA

**Keywords:** Out-of-pocket payment, Catastrophic expenditure, Health insurance, Vietnam

## Abstract

**Background:**

Overcrowding of high-level health facilities is a major concern in a Vietnamese health system. This may increase an economic burden to the households since health insurance is still insufficient in providing financial risk protection. This paper sought to examine the association between the use of high-level health facilities and household-level expenditure status such as out-of-pocket (OOP), and catastrophic expenditure on health, as well as a moderating effect of health insurances in rural and urban districts of Vietnam.

**Methods:**

Data utilized a health system community survey collected between 2015 and 2017 in two districts of Vietnam (one from rural area in northern part, and the other one from urban area in sourthern part). The world Health Organization’s definition of catastrophic expenditure was used. Multivariate tobit and logistic regression were employed for catastrophic expenditure and OOP respectively. Interaction term between health insurance status and visit frequency in high-level facilities was included to investigate the moderating effect of health insurance.

**Results:**

Health insurance status was associated with neither OOP health expenditure nor catastrophic expenditure occurrence, whereas visit frequency of high-level health facilities was strongly associated with both outcomes in both districts(e.g., for catastrophic expenditure, ORs are 1.77 and 1.30 in northern and southern district respecitvely. *P* values are < 0.001). Significant interaction between health insurance status and use of high-level facilities on catastrophic expenditure occurrence was found in Quoc Oai district (OR = 0.68, *p* < 0.05).

**Conclusions:**

The present study demonstrated negative financial impact of utilizing high-level facility on household financial status and weak role of health insurance in decreasing this impact. Multi-faceted approach is called for to mitigate the patient’s financial burden.

**Electronic supplementary material:**

The online version of this article (10.1186/s12913-019-4115-0) contains supplementary material, which is available to authorized users.

## Background

Heavy reliance on out-of-pocket (OOP) health expenditure to finance health care is commonly seen in many low- and middle-income countries. Vietnam is not an exception. Vietnam has remained above the global average in OOP expenditure as a percentage of total health expenditure since 2000, ranking 69th in 2014 of 186 countries [1].

Since Vietnam introduced user fee in 1989 as part of broad market economic reforms known as Doi Moi, hospitals were allowed to recover cost through user fee, requiring people to pay a certain portion of fee for some healthcare services. As a result, the OOP payment as a share of total health expenditure has been always high, ranging from 50 to 70% during 1990s and 2000s [2, 3]. Vietnamese National Health Insurance was introduced in 1989 and began to be implemented in 1992 to help mobilize resources and serve as an appropriate mechanism to mitigate the adverse impact of user fees. In 2008, health insurance law was issued whereby different government programs were merged under a unified national health insurance and free enrollment was offered to the poorer section of population, in an effort to make health insurance the primary mechanism for achieving Universal Health Coverage (UHC) [4]. Since then, Vietnam has made a great stride in expanding social health insurance as one of the leading countries moving toward UHC, with social insurance coverage reaching to 65% of the population in 2011, and 70% in 2013 [5].

Although the proportion of OOP health expenditure declined significantly as a result of increasing population coverage of health insurance, recording about 44.8% by 2010 [6], it was still too steep to be affordable for the population [7]. The concerned government made yet another major effort to implement the “2011-2015 Five-Year Health Sector Plan” with the objectives to 1) increase the public spending on health and 2) improve efficiency through financial reform and allocation methods adjustment. This reform has led to three major changes: 1) giving hospital full autonomy in financial management; 2) revising user fee schedules; and 3) introducing private hospitals, clinics, and pharmacies through the government’s encouragement for public-private partnership in health sector. However, the reform had resulted in decrease in public spending on health, contrary to the intention of the plan, which led to the increase in household OOP share on health from 44.8% in 2010 to 48.8% in 2012 [6].

Several combined factors are driving high OOP health expenditure recently. The first is a phenomenon of overcrowding of hospital-level facilities in Vietnam. A public health system in Vietnam is organized across four scales: national, provincial, district, and commune. National and central hospitals are located in the country’s major urban centers and provide highly-specialized tertiary care for referrals from a provincial hospital. Provincial and district hospitals offer tertiary and secondary care, accepting referrals for more specialized treatment from a lower level. Commune health centers deliver basic primary care and prevention services on an outpatient basis [4]. According to the regulation, insured members can use health services only from the commune health center or district hospital where they are registered. However, limited quality of health care services at the grassroots level reduced patient’s trust for primary care service, leading them to bypass lower level facilities and directly go to higher level facilities. This inefficiency is exacerbated by Vietnamese Social Security (VSS) reimbursement policy where VSS still reimburse for the patients who bypass the referral system, only with a higher copayment rate [4]. Therefore, patients can use high-level facilities still being covered by health insurance, which even facilitate bypass of lower level facilities. Resultant crowding in hospitals increases causes an undue financial burden on patients because hospitals were vested with financial autonomy since 2008 to establish price lists for auxiliary services at their discretion to create supplementary revenues for their recurrent budget which drove prices of un-reimbursed services higher [3]. The second is a provider payment based on fee-for-service (FFS) scheme in Vietnam. Despite the government’s continuous effort to change provider payment method [2], FFS is still a dominant mechanism in Vietnam. FFS incentivizes providers to overprescribe drugs and technology, and/or focus more on only high-profit technology. Preference for high-level facilities under the context of a hospital’s autonomy and FFS payment mechanism has been resulting in high OOP health expenditure for patients. What make matters worse, copayment was re-introduced in 2010 and the government started to charge a significantly higher copayment; 30, 50, and 70% at district, provincial, and central and tertiary hospitals, respectively, when patients bypass lower level facilities while it usually ranged from 5 to 20% otherwise [4].

High OOP may bring about financial catastrophes to households. Health expenditure from household that threatens a household’s financial capacity to maintain its subsistence needs is termed as “catastrophic”. Catastrophic is not necessarily equal to high cost [8]. Even relatively small spending on health can be financially threatening for poor households, leading them to indebtedness, eventually making them fall into poverty [9]. Catastrophic health expenditure is one of the most commonly used indicators globally for measuring extent of burden occurred by medical expenditure for household [10]. Indeed, most of developed countries equipped with adequate financial protection mechanisms whether through health insurance or tax-funded health system, have a relatively low prevalence of catastrophic expenditure.

Minh, et al. (2013) investigated determinants of catastrophic expenditures using the Vietnamese Living Standard Survey (VLSS) dataset across five waves; 2002, 2004, 2006, 2008, and 2010, where insurance status showed significant negative association with occurrence of catastrophic expenditure only in 2004 and 2006 [2]. Minh, et al. (2012) found a positive effect of health insurance on the catastrophic expenditure occurrence based on a 2010 sample from one rural province [11]. Another evidence showing a protective effect of health insurance was gathered by Nguyen, et al. (2013), but only based on patients injured by external cause [12]. On the other hand, one study based on a 2013 dataset of four districts within Hanoi which are mostly urban, showed non-significance in association between insurance status and catastrophic expenditures [13].

Like this, although the argument that frequent use of high-level facilities may lead to high OOP payment and consequential catastrophic expenditure is quite an obvious phenomenon as mentioned above, neither an association between the use of high-level facilities and household-level expenditure status nor moderating effect of health insurance on this association has never been demonstrated empirically in previous studies,

The present study is an effort to fill this knowledge gap. First, we examined associations between frequency of high-level facilities use and household financial outcomes such as catastrophic expenditure and OOP expenditure in both rural and urban districts of Vietnam. Second, we further analyzed whether health insurance status moderate the association between the high-level facilities use and catastrophic expenditures by exploring interactions between health insurance status and the use of high-level facilities.

## Methods

### Data and study setting

The data for the study were obtained from a health system survey conducted in two districts of Vietnam: Quoc Oai district located in northern part of the country between July and September 2015 and Thuan An town located in southeastern part between June 2016 and February 2017). Quoc Oai district, 20 km west of Ha Noi capital, is mostly rural, while Thuan An town of Binh Duong Province, is mostly urban.

Quoc Oai district covers an area of 147 km^2^, including lowlands and mountains and spreads over 20 communes and one town. The number of households and population in Quoc Oai in 2014 was 46,455 and 175,835, respectively. Health care system in Quoc Oai includes district health bureau, district hospital, district center for preventive medicine, and 21 commune health stations. The Thuan An town covers an area of 116 km^2^ and population of 438, 922 as of 2011. There are ten commune health centers, nine of them in urban and one in a rural area.

Health system survey was performed with collaboration of Vietnam Ministry of Health (MoH), Hanoi University of Public Health (HUPH), Hanoi Medical University (HMU), University of Medicine and Pharmacy of Ho Chi Minh City (UMP) of Vietnam, and Seoul National University (SNU) from Korea in an effort to generate scientific evidence that health policymakers and managers can utilize during the process of health system reform in Vietnam [14–16], which is underway to solve emerging health problems occurring from the changes in socioeconomic situation and subsequent epidemiological transitions [17, 18]. The survey covered 16 topics on basic socio-demographic information, disease status, healthcare utilization, health behaviors, pregnancy, mortality, and quality of life (which is only captured for elderly).

### Sample

The survey employed a stratified multi-stage cluster sampling technique. First, the districts were divided into two strata from which 30 clusters were randomly selected. Clusters representing villages were selected by Probability Proportional to Size (PPS) sampling technique to ensure that the number of chosen clusters were proportional to the total population in the district. PPS sampling is especially useful when the sampling units differ greatly in size and also when the sample is drawn in multiple stages [19]. Next, households were selected randomly for each cluster, which yielded 2400 and 1857 households in the northern and southern district, respectively. Finally, one respondent aged 19–60 years was selected from each household. If more than one elderly over 60 years old lived in a household, one of the elderly was selected additionally for respondent, resulting in 2970 and 2155 observations in the northern and southern district, respectively.

Data were collected by face-to-face interviews by trained surveyors under the supervision of a staff team from the Hanoi Medical University, district health center or district hospital. All collected data were electronized using EpiDATA 31(The EpiData Association, Odense, Denmark).

### Outcome measure

Main outcome variables are households’ total OOP health expenditure per year and catastrophic health expenditure occurrence. OOP health expenditure was operationalized as a continuous variable while catastrophic health expenditure was a dummy variable with yes or no. Definition of OOP health expenditure and catastrophic health expenditure suggested by the WHO was used: [20].

#### OOP health expenditure

OOP health expenditure refer to a payment made by the household when they utilize the healthcare service. It includes expenditure incurred for not only direct medical service, but also unofficial expense such as under-the-table payment if incurred. Cost for purchasing alternative and/or traditional medicine and self-treatment are also included whereas, direct non-medical expenditure i.e., a cost for transportation, special nutrition, and health insurance premium were excluded.

#### Household capacity to pay

A household’s capacity to pay means the residual income remaining after basic subsistence needs, which equates to total consumption expenditure of the household. For households that reported food expenditure lower than subsistence spending without counting other non-cash means of food consumption, e.g., food subsidies, coupons, or self-production, non-food expenditure substituted for non-subsistence spending.

#### Household subsistence spending

The household subsistence spending which indicates the minimum amount of money required for basic life in a society was calculated by multiplying a poverty line and house equivalent scale.

#### Poverty line

Poverty line equals to a weighted average of food expenditures of households whose share of food consumption is between the 45th and 55th percentile of the total sample.

#### House equivalent scale

House equivalent scale which has been estimated based on 59 countries’ household survey data was, which equals 0.56 [21].

#### Catastrophic health expenditure

Catastrophic health expenditure was defined as OOP spending for healthcare that equals or exceeds 40% of the household’s capacity to pay or non-subsistence spending and is operated as a binary variable.

### Independent variables

The main independent variables of interest in our study are health insurance status, and frequency of high-level facilities use per year. Health insurance status is binary indicating whether there is at least one health insurance enrollee within household. As for the type of health facilities visited, respondents were asked at which type of health facilities respondents or other family members within household visited for outpatient or inpatient services during the last 12 months. District, provincial, and central hospitals, public or private, were categorized as high-level facilities coded as 1 while all types of health center, private clinics, family doctors and traditional healers were categorized as low level facilities coded as 0.

Other covariates were selected based on evidences available in the previous literatures. As characteristics of the head of the household, gender (male/female), marital status (married/non-married) and education level (primary school graduate or less/high school graduate or less/collage graduate or above) were included while type of residency (rural/urban), household size, having child aged ≦6 (yes/no), having elderly aged ≧ 65 (yes/no), economic status measured by quintile group and total number of chronic and acute diseases any of household member was diagnosed with during the previous 12 months and the previous 3 months respectively were included as household characteristics. Household size, and total number of chronic and acute disease in the household was operated as continuous variable.

### Statistical analysis

Unit of analysis is a household. First, descriptive analyses were undertaken to understand the distribution of respondents, and pattern of OOP health expenditure and occurrence of catastrophic health expenditure according to the categories of independent variables. OOP health expenditure by service type (inpatient, outpatient services and self-treatment) was described additionally.

Since cost data such as OOP expenditure typically exhibit highly skewed distribution due to significant share of observations with zero cost and a minority of observations with extremely high cost, we employed Tobit regression for the analysis with OOP health expenditure as outcome variable [22]. For analysis with the outcome of catastrophic health expenditure, logistic regression was employed.

For each analysis, model 1 includes all independent variables. Then we included interaction term between health insurance status and visit frequency in high-level facilities in model 2.

## Results

Respondents with missing values across any of the variables were excluded. From the sample of Quoc Oai district, 36.2% of households were excluded due to the missing information, of which 29.4% exclusion occurred in the health care utilization-related variables such as type of health facilities used and out of pocket payment. This yielded to 1531 households in the analytic sample. Additional file 1: Table S1 compares the composition of analytical sample and missing observations (Additional file 1: Table S1). In summary, analytical sample has lower proportion of households having at least one child or elderly, slightly poorer, and also lower proportion of households having at least one chronic or acute disease. Taking consideration into this, it is assumed that missing observations are less likely to use the health care service. From the sample of Thuan Anh town, 9.6% of households were excluded due to missing at least one observation across any of the variables, which led to the 1678 household in the final sample.

Table 1 provides descriptive statistics by categories of independent variables. Proportion of households having at least one health insurance enrollees in Quoc Oai district are approximately 30% higher compared to Thuan Anh town. A household in Quoc Oai district used high-level facilities more frequently than a household in Thuan Anh town on average with statistical significance. Proportion of households with at least one child aged less than 6 or with at least one elderly aged more than 65 were slightly higher in Quoc Oai district than Tuan Anh Town in our study samples. Average numbers of acute and chronic diseases per household were statistically higher in Quoc Oai district than Thuan Anh town (1.22 vs.0.99 for chronic disease and 2.65 vs. 2.01 for acute disease, *p* < 0.05).

**Table 1:** Descriptive statistics of study sample

	Quoc Oai				Thuan An			
	N	(%)	OOPE^a^	% of CE	N	(%)	OOPE^a^	% of CE
Having at least one HI enrollee								
No	120	(7.8)	13,303	19.2	633	(37.7)	6727	4.6
Yes	1411	(92.2)	10,877	12.2	1045	(62.3)	7678	6.4
Avg visit freq.of high-level facilities^c^	2.07	(3.02)^a^			1.58	(2.17)^a^		
0	427	(27.9)	4163	4.0	632	(37.7)	2638	2.4
1 ≤	1104	(72.1)	13,684	16.1	1046	(62.3)	10,148	7.7
Having child in HH								
No	836	(54.6)	9545	15.9	1079	(64.3)	7301	6.3
Yes	695	(45.4)	12,903	8.9	599	(35.7)	7352	4.7
Having elderly in HH								
No	1099	(71.8)	8767	10.7	1474	(87.8)	7192	5.6
Yes	432	(28.2)	16,932	17.8	204	(12.2)	8243	6.4
Type of residence (north)								
Plain	1159	(75.7)	11,677	13.8				
Mountainous	372	(24.3)	9146	9.4				
Type of residence (south)								
Rural					63	(3.8)	2899	3.2
Urban					1615	(96.2)	7492	5.8
Wealth quintile								
1	292	(19.1)	10,590	19.5	328	(19.5)	6396	10.4
2	278	(18.2)	19,164	20.9	343	(20.4)	6289	7.0
3	307	(20.1)	9227	13.0	340	(20.3)	5762	3.5
4	327	(21.4)	9073	6.7	337	(20.1)	9704	5.0
5	327	(21.4)	8275	5.5	330	(19.7)	8479	2.7
Marital status of HH head								
Non-married	210	(13.7)	9923	15.2	381	(22.7)	6940	7.1
Married	1321	(86.3)	11,248	12.3	1297	(77.3)	7431	5.3
Education level of HH head								
Illiterate	30	(2.0)	5605	20.0	57	(3.4)	2410	0.0
≦Primary school graduate	411	(26.8)	9446	16.8	469	(27.9)	6842	7.0
≦High school graduate	935	(61.1)	12,790	11.6	1034	(61.6)	7723	5.5
College ≦	155	(10.1)	6058	7.7	118	(7.0)	8052	5.1
Gender of HH head								
Male	1259	(82.2)	11,462	12.2	883	(52.6)	7581	5.5
Female	272	(17.8)	9248	15.1	795	(47.4)	7029	5.9
Number of chronic diseases in HH^c^	1.22	(1.46)^b^			0.99	(1.42)^a^		
0	636	(41.5)	6519	8.2	866	(51.6)	5748	3.8
1 ≤	895	(58.5)	14,233	16.0	812	(48.4)	8996	7.8
Number of acute disease in HH^c^	2.65	(1.91)^b^			2.01	(1.75)^a^		
0	173	(11.3)	7497	11.0	351	(20.9)	5869	4.6
≤ 1	1358	(88.7)	11,478	13.0	1327	(79.1)	7703	6.0
Total average	1531	(100)	11,029	12.7	1678	(100)	7320	5.7
(t-test *p*-value comparing north vs. South)							0.03	0.000

*HI* health insurance, *HH* household, *Avr freq.* average frequency, *CE* catastrophic expenditure

^a^Unit 1000 Vietnamese Dong (≒ 0.04 USD as of Oct 6, 2018)

^b^Mean (SD)

^c^significantly difference in statistical test between north (Quoc Oai) and south (Thuan An)

Average OOP health expenditure per household per year (11,029,000 VND in north vs 7320,000 VND in south (approximately 474 US $ in north vs 314 US $ in south, *p* < 0.05) and catastrophic expenditure occurrence was significantly higher in Northern district (12.7% in north vs. 5.7% in south, *p* < 0.001).

**Fig. 1:**
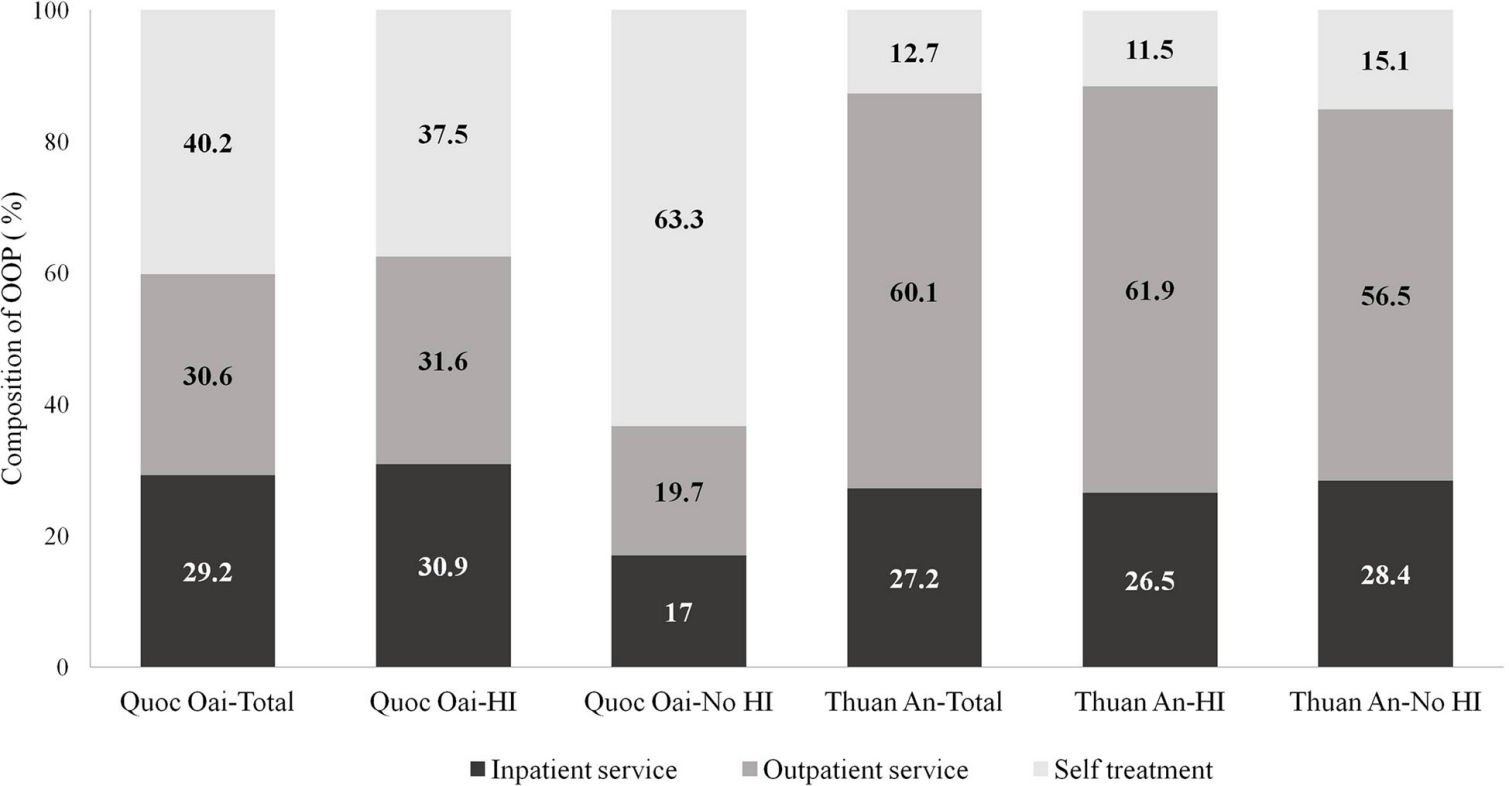
Composition of out-of-pocket health expenditure according to the area and health insurance status

Composition of OOP health expenditure depended on districts. Generally, there was not much difference in the share of OOP health expenditure for inpatient services between two districts (29.2% in the north and 28.2% in the south). On the other hand, proportion of OOP health expenditure spent on purchasing self-treatment was much higher in Quoc Oai (40.2% in the north and 12.7% in the south) while proportion of OOP for outpatient services was more than 30% higher among households in Thuan An town compared to the one in Quoc Oai district (30.6% in the north and 60.1% in the south). Within each district, household without at least one health insurance enrollee spent higher proportion of their total health expenditure on purchasing self-treatment than household with health insurance enrollee, especially in Quoc Oai district with about 26% gap (Fig. 1).

Table 2 represents Tobit regression results on association between predictors and OOP health expenditure. In model 1, having at least one health insurance enrollee did not have any statistically significant association with the amount of OOP health expenditure in both districts, meaning that health insurance does not play a role in decreasing OOP health expenditure. On the other hand, visit frequency in high-level facilities showed strong positive association in both districts. Specifically, one additional visit in high-level facilities was associated with about 1,923,000 VND (about 84 US $) and 2,340,000 VND (about 102 US$) higher in OOP expenditure per year in Quoc Oai district and Thuan An town, respectively. No significant interaction between insurance status and visit frequency in high-level facility on OOP expenditure was found in model 2.

**Table 2:** Tobit regression results for OOP health expenditure

	Quoc Oai		Thuan An		
	Model 1	Model 2	Model 1		Model 2
Having at least one HI enrollee					
No					
Yes	− 7286	−10,474	− 701		−10
Visit freq. in high level facilities	1923^**^	− 308	2340^***^		2663^***^
HH size	− 1623	− 1643	492		490
Having child ≤6 years					
No					
Yes	8999	9000	− 1550		− 1583
Having elderly ≥65 years					
No					
Yes	1053	1009	− 987		− 950
Type of residence (north)					
Plain					
Mountainous	− 2123	− 2220			
Type of residence (south)					
Rural					
Urban			4510^*^		4521^*^
Wealth quintile					
1					
2	8464	8294	−75		−70
3	− 733	− 830	− 1039		− 1104
4	−748	− 908	1813		1743
5	− 3099	− 3212	249		214
Number of chronic disease	6429^***^	6484^***^	1297^***^		1301
Number of acute disease	341	310	22		31
Marital status of HH head					
Non-married					
Married	1099	926	− 426		− 424
Education level of HH head					
Primary school (Illiterate)					
High school	4724	4808	720		716
College ≦	− 1032	− 951	484		582
Gender of HH head					
Male					
Female	− 3391	− 3461	−201		− 225
Insurance x visit freq. in high level facilities		2357			− 475

• Unit: 1000 Vietnamese Dong (≒ 0.04 USD as of Oct 6, 2018)

• *HI* health insurance, *HH* household

• ^*^p ≦ 0.05, ^**^p < 0.01, ^***^p < 0.001

Results on the association with catastrophic expenditure as an outcome variable showed similar picture (Table 3). No statistically significant association between health insurance status and catastrophic expenditure occurrence was found while visit frequency in high-level facilities was positively associated with the odds of catastrophic expenditure both in Northern Quoc Oai district and Southern Thuan An town. One additional visit in high-level facilities increased the odds of catastrophic health expenditure 1.22 and 1.30 times in Quo Oai district and Thuan An town, respectively. A notable difference from the result for OOP expenditure is that a significant interaction was found between visit frequency in high-level facility and health insurance status. In other words, households with at least one health insurance enrollee are less likely to experience catastrophic health expenditure caused by high-level facilities use compared to households without any health insurance enrollees. However, this effect appeared only in Quoc Oai district.

**Table 3:** Logistic regression results for catastrophic expenditure

	Quoc Oai		Thuan An	
	Model 1 (OR)	Model 2 (OR)	Model 1 (OR)	Model 2 (OR)
Having at least one HI enrollee				
No				
Yes	0.87	1.60	1.18	1.49
Visit freq. in high level facilities	1.22^***^	1.77^***^	1.30^***^	1.39
HH size	0.73^***^	0.73^***^	0.88	0.88
Having child ≤6 years				
No				
Yes	1.06	1.08	0.72	0.71
Having elderly ≥65 years				
No				
Yes	1.86^**^	1.84^**^	0.63	0.64
Type of residence (north)				
Plain				
Mountainous	0.65	0.66		
Type of residence (south)				
Rural				
Urban			2.21	2.22
Wealth quintile				
1				
2	0.92	0.92	0.65	0.66
3	0.53^**^	0.52^**^	0.30^**^	0.29^**^
4	0.27^***^	0.27^***^	0.41^**^	0.40^**^
5	0.22^***^	0.22^***^	0.22^***^	0.21^***^
Number of chronic disease	1.16^*^	1.15^*^	1.25	1.25
Number of acute disease	0.87^**^	0.87^*^	0.98	0.99
Marital status of HH head				
Non-married				
Married	1.28	1.27	0.80	0.81
Education level of HH head				
Primary school (Illiterate)				
High school	0.85	0.86	0.97	0.97
College ≦	0.66	0.66	0.88	0.93
Gender of HH head				
Male				
Female	0.86	0.89	0.94	0.93
Insurance x visit freq. in high level facilities		0.68^*^		0.91

• *HI* health insurance, *HH* household

• ^*^p ≦ 0.05, ^**^p < 0.01, ^***^p < 0.001

Among other covariates including household and household head characteristics, only the number of chronic diseases was common factor that was significantly associated with OOP health expenditure in both districts (Table 2). On the other hand, catastrophic expenditure showed a significant association with several covariates. Household size, having elderly aged more than 65 in household, and the number of chronic and acute diseases were significantly associated with the catastrophic expenditure occurrence in Northern Quoc Oai district. In addition, the level of wealth showed a negative gradient with the catastrophic expenditure occurrence in both districts, meaning that wealthier households were less likely to experience catastrophic health expenditure.

## Discussion

OOP health expenditure and catastrophic health expenditure are key indicators reflecting how well financial protection mechanisms are functioning in a society [20]. The present study demonstrated negative financial impact of utilizing high-level facility on household financial status and weak role of health insurance in decreasing this impact. Specifically, health insurance status was associated with neither OOP health expenditure nor catastrophic expenditure occurrence, whereas visit frequency of high-level facilities was strongly associated with both outcomes in a northern rural district (Quoc Oai) and a southern urban district (Thuan An Town). On the other hand, a significant interaction between health insurance status and use of high-level facilities on catastrophic expenditure occurrence was found in a rural district (Quoc Oai), which is a favorable sign that having health insurance in household can mitigate the possible negative effect that visit in high-level facilities might have on catastrophic expenditure occurrence.

Our descriptive statistics showed disparity in patterns of OOP health expenditure and prevalence of catastrophic expenditure between rural district of northern part and urban town of southern part in Vietnam. OOP health expenditure and prevalence of catastrophic expenditure were higher in rural district (12.7% in rural vs. 5.7% in urban). In previous studies which targeted urban Hanoi city, estimated prevalence of catastrophic expenditure were 10 and 6.6% in slum and non-slum area, respectively, in 2013 [13]. Another study which had examined one rural district estimated 14.6 and 4.2% in the prevalence of catastrophic expenditure for households with and without at least one member having chronic disease each in 2010 [11]. Considering the results from the previous studies and the present study, it seems that there has been little improvement in financial protection mechanism in Vietnam.

Rural area has consistently shown a higher prevalence of catastrophic expenditure in previous studies in other developing countries as well as in Vietnam [2, 23, 24]. In our study, both the descriptive statistics and main analyses might present explanations for a higher prevalence of catastrophic expenditure in rural area. Descriptive statistics revealed that population composition of a northern rural (Quoc Oai) district is more unfavorable than the one in a southern urban district (Thuan Anh town). Specifically, average number of chronic or acute disease per household, proportion of households having at least child less than 6 years old and elderly older than 65 years old were higher in northern rural (Quoc Oai) district with statistical significance, suggesting that medical need might be higher in rural area than urban district. More frequent visits to high-level facilities in Quoc Oai district than in Than Anh town could result from these conditions, although we cannot rule out the possibility that some of them might have been unnecessary visit. One favorable finding is that the proportion of household with more than one health insurance enrollee was much higher in rural Quoc Oai district than in urban Thuan Anh town. This might be because Vietnam health insurance law issued in 2008 made enrollment of all the vulnerable population groups such as children under 6 years old, ethnic minorities, and financially disadvantaged people in compulsory health insurance mandatory, resulting in higher enrollment rate in rural area where there are larger child and elderly populations and lower socio-economic status than urban area.

If health insurance serves its purpose of financial protection properly, the prevalence of catastrophic expenditure would not have been high despite the unfavorable population composition. However, main result from the present study showed non-significant association of having health insurance with both OOP health expenditure and catastrophic expenditure occurrence in both rural and urban areas, signifying that health insurance does not lessen the burden from OOP payment or to stave off catastrophic expenditure.

Null or modest effect of insurance on reducing OOP health expenditure or catastrophic expenditure occurrence in Vietnam was consistently found in previous studies although it is somewhat outdated [13, 25, 26]. This might be because continuous effort to improve social health insurance for the last decade has mainly focused on expanding coverage to a larger fraction of the population rather than deepening the coverage.

The strong association found between visit frequency of high-level facilities and catastrophic expenditure occurrence as well as OOP health expenditure deserves great attention from the Vietnamese society because bypassing primary health care and crowding into high-level hospital is one of the biggest sources of inefficiency in health care throughout the whole country currently. As mentioned before, a mixture of factors such as financial autonomy granted to hospitals, dominance of FFS payment system, and higher co-payment rate charged to patients who visit high-level facilities bypassing primary care may explain the positive association between the frequency of high-level facilities use and OOP health expenditure and catastrophic expenditure. Unnecessary utilization of high-level facilities needs to be removed not only for macro-efficiency but also for keeping individual patients from catastrophic expenditure.

All of these factors such as higher medical demand, higher use of high-level facility in rural area and insufficient functioning of social health insurance in financial protection are assumed to be contributing to higher prevalence of catastrophic expenditure in rural Quoc Oai district. Although cross-sectional characteristics of our data did not allow us to examine the effect of catastrophic expenditure on impoverishment, it can be expected that households in rural Quoc Oai district have been more likely to be impoverished considering the lower socioeconomic status in the rural district.

On the other hand, we found one favorable evidence on health insurance benefit that there was a significant difference in the association between visit frequency of high-level facilities and catastrophic expenditure occurrence according to health insurance status in the northern rural district. This means that the likelihood of catastrophic expenditure caused by using high-level facilities was lower among the households with at least one health insurance enrollee than their counterparts. While health insurance did not show a direct effect in decreasing catastrophic expenditure overall, it was shown to have a protective effect in decreasing catastrophic expenditure caused by using high-level facilities. Frequent utilization of high-level facilities can be linked to a high level of expenditure on medical cost. Although coverage depth of current social insurance is not high enough to protect the people from experiencing catastrophic expenditure overall, it might be helpful for reducing burden at least for high-cost patients such as frequent users of high-level facilities. The reason that this moderating effect appeared only in a rural area might be explained as follows. Given the fact that the number of chronic and the number of acute disease per household is higher in a rural area, total medical cost as well as out of pocket payment would be higher in a rural area. Therefore, benefit in reducing the economic burden from social health insurance caused by using high-level facilities would be bigger in a rural area.

There is an additional finding to note. Partitioning of the OOP health expenditure by service type revealed that the proportion of self-treatment among total OOP health payment was higher by almost 29% in the rural Quoc Oai district, and the same percentage among total OOP was spent more on inpatient service in southern Thuan Anh town. Self-treatment is defined as the use of medicine by an individual’s choice without professional advice [27]. Although the main driver for a high share of self-treatment in Vietnamese context needs to be identified in future studies, individuals’ concern over the expenses incurred by using formal health services (possibly due to insufficient financial protection scheme) has been recognized as one of the factors in the previous study [28]. Self-treatment can pose a serious health risk due to high rate of irrational or incorrect use of drugs [29]. There is a need for a government effort to bring those who chose self-medication on the economic ground into the benefit of the health insurance system as soon as possible.

Regarding other covariates associated with catastrophic expenditure and OOP health expenditure, the size of the household was negatively associated with catastrophic expenditure, which is consistent with previous studies [30, 31]. A larger family may be able to spread financial burden to the household member through mobilizing more resources from broader social networks at the times of needs. Findings that the presence of elderly people within the household was a strong predictor of catastrophic expenditure have also demonstrated in Minh, et al’s result [2]. This result should not be overlooked considering that Vietnam has no proper safety net for the elderly’s health while Vietnam is undergoing rapid population aging [32, 33].

Major strength of the present study is that we were able to examine impact of high-level facility use on financial status empirically and potential mitigation effect of national health insurance in it. Another strength is that our study design allows us to compare the rural and urban area based on the comparably selected samples. On the other hand, a few limitations of this study should not be overlooked. First, there was substantial level of missing information in variables related the healthcare utilization in Quoc Oai dataset. Supposedly, this might have not distorted the result considering a similar prevalence of catastrophic expenditure between the present and previous studies. However, we still cannot be free from concern about bias. Second, the self-reported nature of the data can cause measurement error in our sample. Although medical expenditure information was obtained from the receipt of service utilization if it was available, it still cannot rule out potential biases. Third, since the study design is cross-sectional, interpretation should be limited only to the association rather than causality. Third, the data for our study were drawn only from a single district each from northern and southern regions, and are therefore not fully representative of Vietnam. Therefore, care should be given for generalizing the result to other parts of the country.

## Conclusion

Increasing people’s demand for healthcare and deteriorating quality of primary care are inducing people to prefer high-level facilities, bypassing primary service. In addition, health insurance which is expected to ensure financial protection still has significant challenges. The resulting financial threat is more serious in the poor rural area than the urban area. Findings from this study are calling attention to the need for a multi-faceted approach to mitigate the patients’ financial burden caused by healthcare service use, such as improving service at the primary care level, strengthening health insurance and diverting self-treating population into seeking proper health service. Sophisticated designs of copayment of health insurance that deepen cost coverage of health insurance, but not induce overuse from moral hazard, would also be necessary to ensure the people from the catastrophic payment due to rational medical service utilization.

## Additional file

Additional file 1: **Table S1.** Comparison of characteristics between analytic sample and dropped sample (%). Results from comparison of characteristics between analytic sampld and dropped sample due to missing information. (DOCX 99 kb)

## Abbreviations

FFS: Fee-for-service
HMU: Hanoi Medical University
HUPH: Hanoi University of Public Health
OOP: Out-of-pocket
PPS: Proportional to Size
SNU: Seoul National University
UHC: Universal Health Coverage
UMP: Ho Chi Minh University of Medicine and Pharmacy
VLSS: Vietnamese Living Standard Survey
VND: Vietnamese Dong
