# Supplementary material for: Use of high-level health facilities and catastrophic expenditure in Vietnam: can health insurance moderate this relationship?
Source: BMC Health Serv Res. 2019 May 21;19:318. doi: 10.1186/s12913-019-4115-0 (PMC6528376; doi:10.1186/s12913-019-4115-0)
Supplement: Supplementary file 1 — Table S1. Comparison of characteristics between analytic sample and dropped sample (%). Results from comparison of characteristics between analytic sampld and dropped sample due to missing information. (DOCX 99 kb) [file 12913_2019_4115_MOESM1_ESM.docx]

Table S1. Comparison of characteristics between analytic sample and dropped sample (%)

|  | Analytical sample | Dropped sample | Significance |
| --- | --- | --- | --- |
| Having at least one HI enrollee |  |  |  |
| No | 7.8 | 14.4 | *** |
| Yes | 92.2 | 85.6 |  |
| Having child in HH |  |  |  |
| No | 54.6 | 99.5 | *** |
| Yes | 45.4 | 0.5 |  |
| Having elderly in HH |  |  |  |
| No | 71.8 | 89.3 | *** |
| Yes | 28.2 | 10.7 |  |
| Type of residence(north) |  |  |  |
| Plain | 75.7 | 60.5 | *** |
| Mountainous | 24.3 | 39.5 |  |
| Wealth quintile |  |  |  |
| 1 | 19.1 | 21.9 | non-significant |
| 2 | 18.2 | 23.0 | ** |
| 3 | 20.1 | 19.7 | *** |
| 4 | 21.4 | 17.6 | * |
| 5 | 21.4 | 17.7 | * |
| Marital status of HH head |  |  |  |
| Non-married | 13.7 | 16.3 |  |
| Married | 86.3 | 83.7 |  |
| Education level of HH head | | | |
| Illiterate | 2.0 | 2.0 | non-significant |
| ≦Primary school graduate | 26.8 | 26.6 | non-significant |
| ≦High school graduate | 61.1 | 63 | non-significant |
| College ≦ | 10.1 | 8.4 | non-significant |
| Gender of HH head |  |  |  |
| Male | 82.2 | 80.8 | non-significant |
| Female | 17.8 | 19.2 |  |
| N of chronic diseases in HH^**^ | 1.46^a^ | 0.69 |  |
| 0 | 41.5 | 61.3 | *** |
| 1 ≤ | 58.5 | 38.7 |  |
| N. of acute disease in HH^**^ | 1.91 ^a^ | 1.92 |  |
| 0 | 11.3 | 20.3 | *** |
| 1 ≤ | 88.7 | 79.7 |  |

^a^ : Mean (SD) / HI : health insurance /HH: household / ^*^ *p* ≦ 0.05, ^**^ *p* < 0.01, ^***^ *p* < 0.001
